# Supplementary material for: Cycloartenyl Ferulate Is the Predominant Compound in Brown Rice Conferring Cytoprotective Potential against Oxidative Stress-Induced Cytotoxicity
Source: Int J Mol Sci. 2023 Jan 3;24(1):822. doi: 10.3390/ijms24010822 (PMC9821627; doi:10.3390/ijms24010822)
Supplement: Supplementary file 1 [file ijms-24-00822-s001.zip › ijms-2063703-supplementary.pdf]

Table S1. Free radical scavenging capacity of the major phytochemicals in the brown rice.[a]

Rice Sample	αT (nmol TE/g rice)	γT (nmol TE/g rice)	αT3 (nmol TE/g rice)	γT3 (nmol TE/g rice)	FA (nmol TE/g rice)	CAF (nmol TE/g rice)
BR	25.76±2.97	5.08±0.53	24.79±1.99	28.36±2.20	N.D [b]	26.37±2.39

[a] Results are expressed as mean ± SD. (n=4).

[b] Not detected.

Table S2. Trolox equivalent antioxidant capacity (TEAC) for the major phytochemicals in the brown rice.[a]

Standard Compounds	αT	γT	αT3	γT3	FA	CAF
TEAC	0.85±0.10	0.78±0.06	0.73±0.01	0.73±0.02	0.49±0.03	0.26±0.02

[a] Results are expressed as mean ± SD. (n=3).

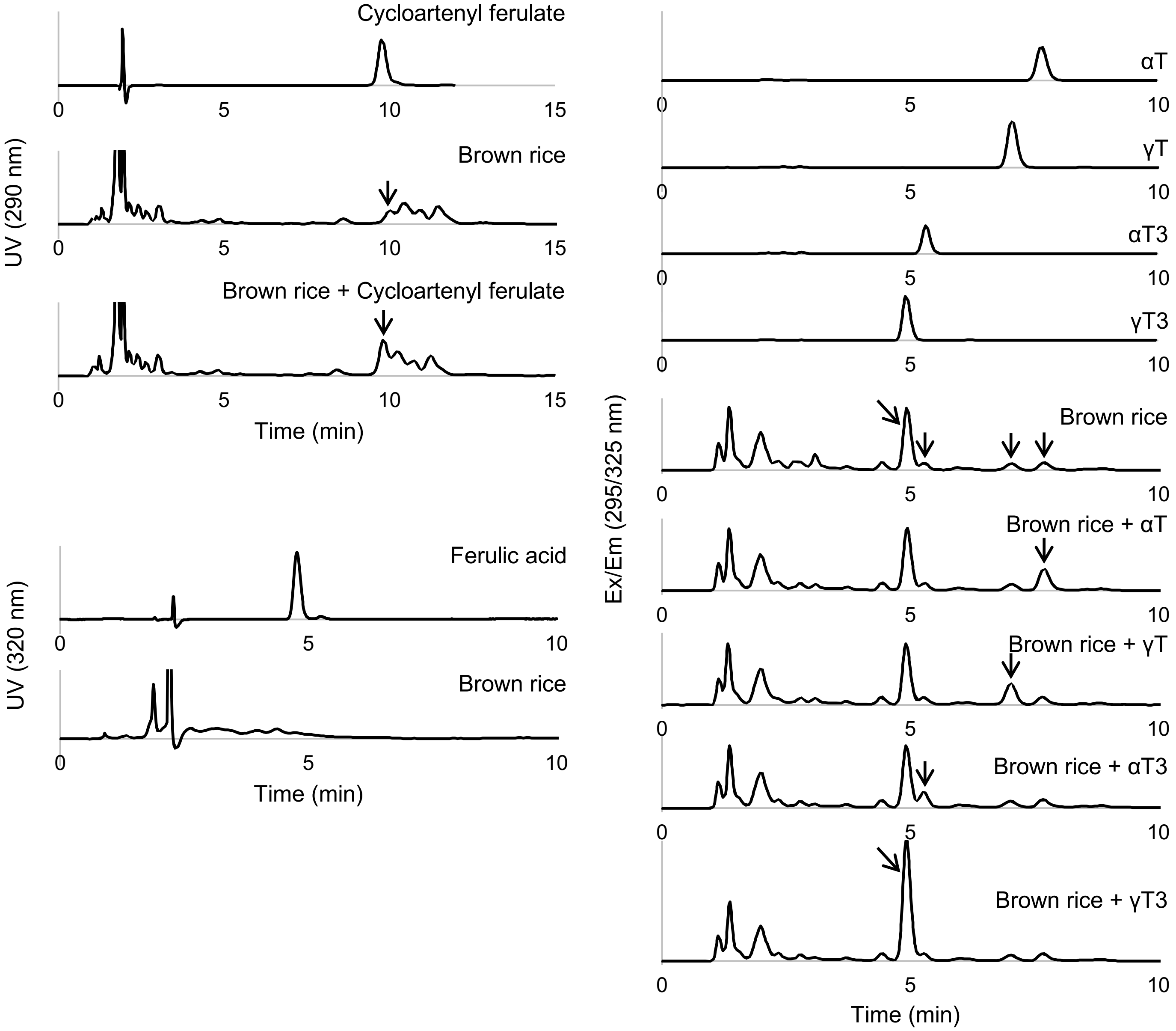

Figure S1. Representative HPLC chromatograms of cycloartenyl ferulate, vitamin E derivatives, ferulic acid and their co-chromatography.
